# The *Archipelago* Ubiquitin Ligase Subunit Acts in Target Tissue to Restrict Tracheal Terminal Cell Branching and Hypoxic-Induced Gene Expression

**DOI:** 10.1371/journal.pgen.1003314

**Published:** 2013-02-14

**Authors:** Nathan T. Mortimer, Kenneth H. Moberg

**Affiliations:** Department of Cell Biology, Emory University School of Medicine, Atlanta, Georgia, United States of America; The University of North Carolina at Chapel Hill, United States of America

## Abstract

The *Drosophila melanogaster* gene *archipelago* (*ago*) encodes the F-box/WD-repeat protein substrate specificity factor for an SCF (Skp/Cullin/F-box)-type polyubiquitin ligase that inhibits tumor-like growth by targeting proteins for degradation by the proteasome. The Ago protein is expressed widely in the fly embryo and larva and promotes degradation of pro-proliferative proteins in mitotically active cells. However the requirement for Ago in post-mitotic developmental processes remains largely unexplored. Here we show that Ago is an antagonist of the physiologic response to low oxygen (hypoxia). Reducing Ago activity in larval muscle cells elicits enhanced branching of nearby tracheal terminal cells in normoxia. This tracheogenic phenotype shows a genetic dependence on *sima*, which encodes the HIF-1α subunit of the hypoxia-inducible transcription factor dHIF and its target the FGF ligand *branchless (bnl)*, and is enhanced by depletion of the *Drosophila* Von Hippel Lindau (*dVHL*) factor, which is a subunit of an oxygen-dependent ubiquitin ligase that degrades Sima/HIF-1α protein in metazoan cells. Genetic reduction of *ago* results in constitutive expression of some hypoxia-inducible genes in normoxia, increases the sensitivity of others to mild hypoxic stimulus, and enhances the ability of adult flies to recover from hypoxic stupor. As a molecular correlate to these genetic data, we find that Ago physically associates with Sima and restricts Sima levels *in vivo*. Collectively, these findings identify Ago as a required element of a circuit that suppresses the tracheogenic activity of larval muscle cells by antagonizing the Sima-mediated transcriptional response to hypoxia.

## Author Summary

Cells in multicellular animals must adapt to changing environmental conditions in order to ensure survival of the larger organism. One key challenge they face is fluctuation in the availability of dissolved oxygen. As cells get low on oxygen, they respond by turning on a program of gene expression that helps them survive. The key to this program is a protein, called HIF-1α in humans and Similar (or Sima) in the fruit fly *Drosophila melanogaster*, that is kept inactive in normoxia but is activated in hypoxia. The mechanisms responsible for this switch are not completely understood. In this study, we present genetic and molecular evidence that a component of the protein degradation machinery called Archipelago is required to keep Sima inactive in developing muscle cells and that genetically removing Archipelago makes these cells “think” they are hypoxic. This finding and the data that support it provide new insight into genetic circuits that cells use to control their response to changing oxygen levels and suggest that defects in oxygen homeostasis may contribute to cancerous disease states associated with loss of the human equivalent of Archipelago called Fbw7.

## Introduction

Metabolically active tissues require an adequate supply of dioxygen (O_2_) for metabolic production of ATP by aerobic glycolysis and as a necessary substrate in a variety of enzymatic reactions (reviewed in [1]). Consequently, cells in metazoan organisms have evolved a conserved hypoxia-response mechanism that senses low O_2_ (or hypoxia) and modulates cellular metabolism and signaling in response to this environmental challenge. Activation of this adaptive mechanism results in changes in transcription that allow organisms to adapt to O_2_ conditions that might otherwise be incompatible with normal development and homeostasis (reviewed in [2]). In most metazoans, these changes include elevated expression of factors involved in oxygen-independent ATP production, increased expression of oxygen-carrying hemoglobin-like molecules and increased branching of O_2_-carrying tubular organs, the net effect of which is to reduce overall O_2_ demand and increase O_2_ delivery.

Molecular mechanisms through which changes in O_2_ concentration alter metabolism and drive increased tubular branching are conserved through the metazoan tree to include invertebrates like the fruit fly *Drosophila melanogaster* (reviewed in [3]). A key element of this mechanism is the hypoxia-inducible factor-1 [4]–[7] (HIF-1 or *Drosophila* HIF [dHIF] in flies), which is a heterodimeric transcription factor composed of an oxygen-regulated HIF–1α subunit and a constitutively expressed HIF–1β subunit. In *Drosophila* these subunits are respectively encoded by the *similar* (*sima*) [8], [9] and *tango* (*tgo*) [10]–[12] genes. The HIF-1/dHIF heterodimer is required for cellular adaptation to hypoxic conditions [4]–[7] and is regulated mainly at the level of HIF–1α stability [reviewed in 13]). In normoxic conditions, HIF–1α is hydroxylated at conserved proline residues by the 2-oxoglutarate/Fe(II)-dependent prolyl-4-hydroxylase family member HIF prolyl hydroxylase (HPH) [14], [15]. Prolyl-hydroxylation of HIF–1α facilitates binding with the von Hippel Lindau (VHL) E3-ubiquitin ligase subunit, and subsequent polyubiquitination and proteasome-dependent degradation of HIF-1α [16]–[20]. *Drosophila* Sima is controlled by a well-conserved version of this pathway involving the HPH homolog *fatiga* (*fga*), and the *Drosophila* VHL homolog, *dVHL* [14], [21]–[24]. Because the HPH enzymatic activity is dependent upon the availability of oxygen [14], [15], the HPH/VHL pathway effectively functions as a sensor of cellular oxygen levels, allowing HIF–1α/Sima stabilization only in hypoxic conditions and preventing HIF activity in normoxic cells [reviewed in 2]. Mutations in the *VHL* gene stabilize HIF-1α and are associated with a dominantly inherited hereditary cancer syndrome in humans that predisposes to a variety of malignant and benign tumors of the eye, brain, spinal cord, kidney, pancreas, and adrenal glands [25]. Excess HIF-1α can promote several important aspects of cancer biology, including the metabolic switch to anaerobic glycolysis characteristic of tumor cells [i.e. the Warburg effect; 26], neoangiogenesis, and increased tumor metastasis [reviewed in 13], [27], [28].

The invertebrate response to hypoxia mirrors key features of the mammalian hypoxic response [3], [29], [30]. Hypoxia stabilizes Sima and induces expression of genes that include homologs of mammalian HIF targets, such as *lactate dehydrogenase* (LDH) [31]. Hypoxic treatment of flies also produces physiological changes reminiscent of the mammalian hypoxic response [32], including altered metabolism and reduced oxygen consumption [33]–[36]. Adult *Drosophila* respond to hypoxia by entering into state of stupor characterized by low or undetectable neurological activity that allows them to tolerate extended periods of low oxygen [34], and recovery from this state is dependent upon genes necessary for survival in low-oxygen conditions [31]–[33], [35]. Hypoxia also induces a neoangiogenesis-like process in *Drosophila* involving increased branching of the tracheal system, an open network of interconnected, epithelial tubes that duct gases in and out of the animal [reviewed in 37]. *Drosophila* larvae reared in chronic hypoxia show increased branching of cells at the tip of each tracheal branch termed ‘terminal tip’ cells, whereas those raised in chronic hyperoxia show a reciprocal decrease in the extent of terminal branch elaboration [22], [38]. This increased larval tracheal branching in low O_2_ involves the FGF receptor homolog *breathless* (*btl*) [39] and the FGF ligand *branchless* (*bnl*) [40]: hypoxic exposure results in a *sima*-dependent increase in expression of *btl* in tracheal cells and *bnl* in peripheral oxygen-deficient tissues [22], [38]. Bnl then acts on tracheal terminal tip cells, which express Btl [41], [42], to induce fine tubular extensions that project toward Bnl-expressing cells. These terminal branches serve as the primary site of gas exchange between the tracheal system and internal tissues. When the oxygen demand is met, Bnl and Btl expression decreases, thereby limiting hypoxia-induced tracheal growth. This oxygen responsiveness allows for growth of tracheal terminal branches specifically to localized areas of hypoxia in order to shape the mature tracheal architecture and to increase oxygen-delivery capacity in hypoxic conditions.

In addition to the oxygen-dependent HPH/VHL pathway, mammalian HIF-1 is regulated by VHL-independent mechanisms that are incompletely understood [43], [44]. Recent studies have linked HIF–1α turnover to phosphorylation by the GSK3ß kinase and subsequent binding of the ubiquitin ligase subunit Fbw7 [45], [46], which is a sequence and functional ortholog of the *Drosophila* Archipelago (Ago) protein. Intriguingly *Drosophila* Ago binds and stimulates turnover of the Trachealess protein (Trh), which is a Sima/HIF-1α sequence homolog, in embryonic tracheal cells [47]. Genetic data show *ago* and *dVHL* also coregulate oxygen-sensitivity in the developing embryonic tracheal arbor [48].

In light of these connections, we have tested the requirement for *ago* in oxygen-sensitive stages of larval tracheal development and find evidence that *ago* is an antagonist of dHIF during the larval stage. Genetic manipulations that reduce *ago* function within post-mitotic larval muscle cells lead to a *sima*-dependent increase in the branching of nearby terminal cells. This phenotype is not suppressed by a *trh* allele that suppresses branch defects in *ago* mutant embryonic tracheal cells [47], but rather correlates with elevated expression of the Sima-induced gene *bnl* expression in larval muscle cells and genetic dependence on *bnl*. At an organismal level, reducing *ago* activity results in constitutive expression of some dHIF target genes in normoxia, increases the sensitivity of others to mild hypoxic stimulus, and allows adult flies to recover more rapidly from hypoxic stupor than normal flies. Significantly, non-cell autonomous effects of *ago* and *dVHL* alleles on terminal branching are synergistic, suggesting that the Ago and dVHL proteins co-regulate dHIF. Consistent with this, Ago protein can be found in a complex with Sima in larval extracts and loss of Ago elevates Sima levels in peripheral tissues. Collectively these findings define an important role for Ago as a required antagonist of the Sima-dependent hypoxic response during the larval stage of *Drosophila* development.

## Results

### Loss of *ago* results in increased branching of tracheal terminal cells

**Figure 1:**
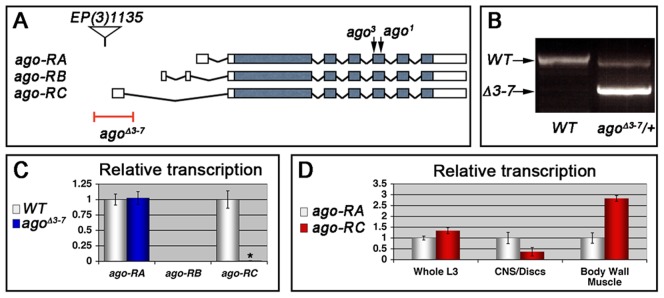
The *ago* ^Δ*3–7*^ allele specifically deletes the *ago-RC* transcript, which is enriched in body wall muscle cells. (A) Graphical representation of the *ago* genomic locus illustrating the three *ago* transcripts and the alleles used in this study. (B) Genomic PCR to confirm the *ago* ^Δ*3–7*^ deletion. (C) Quantification of the relative transcription of the three *ago* transcripts in larvae in the indicated genotypes by quantitative real-time PCR (qRT-PCR). (* p<2.7×10^−5^ relative to control). (D) Tissue specific qRT-PCR of the indicated *ago* transcripts to assay relative abundance in the indicated tissues.

Heterozygosity for a null allele of *ago* sensitizes the *Drosophila* embryonic tracheal system to mild hypoxia [48]. To determine whether *ago* is also involved in hypoxia responsiveness in the subsequent larval stage, it was necessary to generate an allele of *ago* that allowed development beyond the late embryonic lethality associated with *ago* null alleles [49]. This was achieved by transposase-mediated imprecise excision of *EP(3)1135*, a P-element located 16 base pairs (bp) upstream of the *ago* genomic locus (Bloomington Drosophila Stock Center [BDSC]) that behaves genetically as a weak *ago* hypomorph. Excision of *EP(3)1135* produced a 603 bp deletion removing the first exon of the *ago*-*RC* transcript (Figure 1A–1B) that was designated *ago^Δ3^* ^–*7*^. The effect of *ago^Δ3–7^* on patterns of *ago* transcription was determined by quantitative real-time PCR (qRT-PCR). Of the three predicted *ago* transcripts (*ago-RA*, *ago-RB*, and *ago-RC*) only *RA* and *RC* are detected in whole larvae (Figure 1C). Consistent with the location of the deletion in the *ago^Δ3–7^* allele, the *ago-RC* transcript is specifically absent in *ago^Δ3–7^* mutant larvae while expression of the *ago-RA* transcript is unaffected. Notably, the *ago-RA* and *RC* transcripts display inverse expression patterns: *ago-RA* is approximately 3-fold more abundant than *ago-RC* in imaginal discs and larval brain and ventral nerve cord, but *ago-RC* is 3-fold more abundant than *ago-RA* in filleted larval body wall preparations (Figure 1D). The *ago^Δ3–7^* allele is thus a tissue- and transcript-specific allele that primarily reduces *ago* expression in peripheral tissues such as body wall muscle.

**Figure 2:**
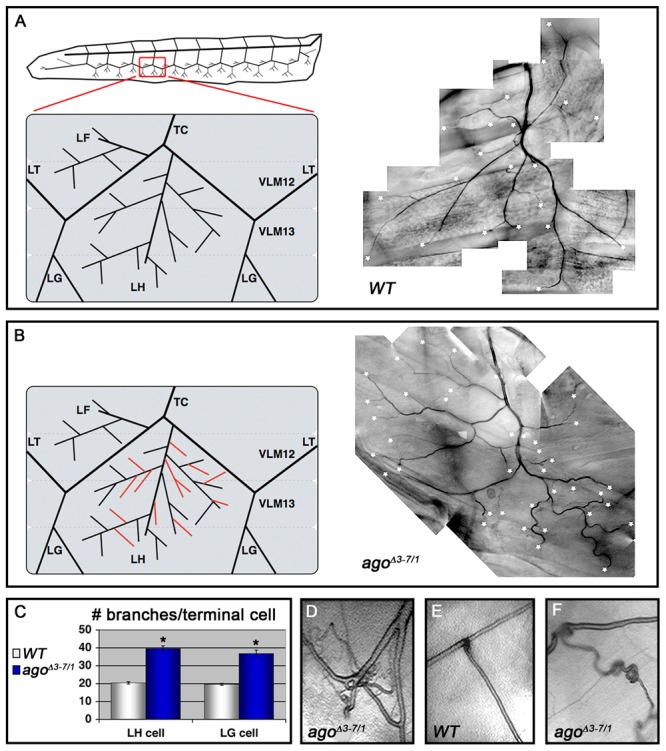
*ago* ^Δ*3–7/1*^ larvae display a wide range of tracheal terminal branch phenotypes. (A,B) Schematic (left) and representative photomicrograph showing branching of LH lateral terminal cells in control (A) and *ago* ^Δ*3–7/1*^ (B) larvae. Branch termini are indicated with asterisks. (C) Quantification of branch number per LH (left) and LG (right) terminal cell in the indicated genotypes (*p<0.001 relative to control). (D) *ago* ^Δ*3–7/1*^ larva displaying terminal branch tangling. (E,F) Ganglionic tracheal branches in control (E) and *ago* ^Δ*3–7/1*^ (F) larvae. Ringlet-shaped branches and tangles occur in approximately 25% of *ago* ^Δ*3–7/1*^ larvae.

Approximately 49% of *ago^Δ3^* ^–*7*^ homozygotes or *trans*-heterozygotes in combination with the null alleles *ago* ^1^ and *ago* ^3^ die as pupae (Table 1) and the remainder die as late 2^nd^ and 3^rd^ instar larvae (data not shown). Those that live to late 3^rd^ instar show tracheal phenotypes (Figure 2 and Table 2). The most prevalent of these phenotypes is an approximate doubling of the number of cytoplasmic branches elaborated from multiple subtypes of terminal cells, including those found along the lateral trunk that serve to oxygenate the ventrolateral body wall muscles: LH cell terminal branching increases from 20.4±0.64 branches (n = 33) in control larvae to 39.5±1.59 branches (n = 34) in *ago^Δ3–7/1^* larvae (p = 2.6×10^−16^) (Figure 2A–2C and Table 2), and LG cell terminal branching increases from 19.6±0.54 branches (n = 33) in control larvae to 36.8±1.94 branches (n = 31) in *ago^Δ3–7/1^* larvae (p = 9.5×10^−13^) (Figure 2C). Notably, the magnitude of these increases in terminal cell branch number is similar to that seen in larvae grown in hypoxic conditions [22], [38]. Loss of *ago* function also causes additional tracheal branch phenotypes in approximately 25% of larvae, including the appearance of terminal branch tangles (Figure 2D) and the development of ‘ringlet’-shaped ganglionic branches (Figure 2F), which resemble phenotypes seen in hypoxic larvae or those in which Sima is activated by genetic disruption of the Fga/dVHL regulatory pathway [22]. Given the transcript- and tissue-specific nature of the *ago^Δ3–7^* allele, these tracheal phenotypes support the hypothesis that *ago* has a non-autonomous role in in restricting terminal branching.

**Table 1:** Pre-pupal lethality of *ago* allele combinations.^a^

Genotype	% observed (± SEM)	n
*ago^Δ3–7^*	49.8±3.26	983
*ago^Δ3–7^*, *sima^07607^/+*	77.4±7.61^b^	1688
*ago^Δ3–7/1^*	34.4±4.37	1056
*ago^Δ3–7/1^*, *sima^07607^/+*	67.7±5.17^c^	1680
*ago^Δ3–7/3^*	43.2±5.25	1153
*ago^Δ3–7/3^*, *sima^07607^/+*	71.0±7.82^d^	878

a Calculated as # observed/# expected.

p<0.05 relative to

b *ago^Δ3–7^*,

c *ago^Δ3–7/1^*,

d *ago^Δ3–7/3^*.

**Table 2:** Number of tracheal terminal branches per LH cell.

Genotype	# branches/LH cell (± SEM)	n
*wt*	20.4±0.64	33
*ago^Δ3–7/1^*	39.5±1.59^a^	34
*ago^Δ3–7/1^*,*sima^07607^/+*	29.0±1.48^b^	34
*ago^Δ3–7/1^*,*bnl^P1^/+*	28.4±1.80^b^	29
*ago^Δ3–7/3^*	42.2±2.94^a^	6
*ago^Δ3–7/3^*,*sima^07607^/+*	26.1±1.62^c^	16

p<1.0×10^−4^ relative to

a *wt*,

b *ago^Δ3–7/1^*, or

c *ago^Δ3–7/3^*.

### *ago* acts non-autonomously to restrict post-embryonic tracheal branching

**Figure 3:**
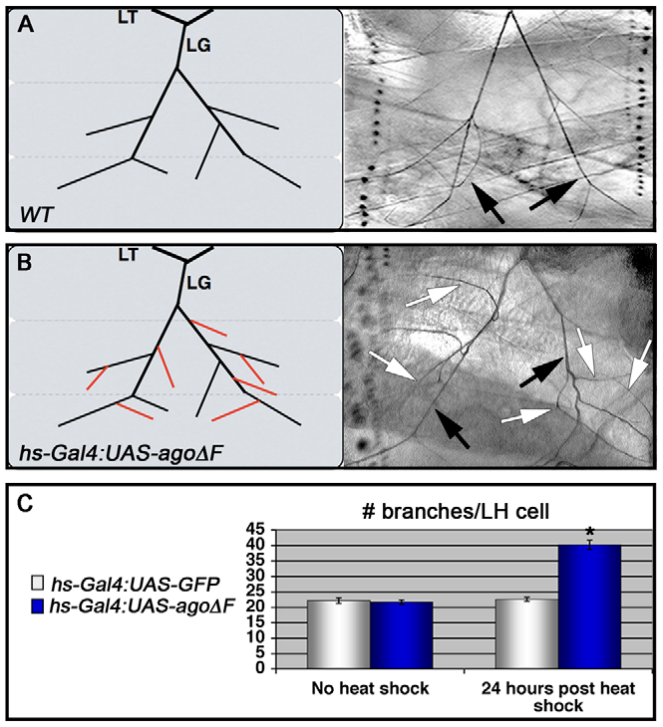
Heat shock induction of *ago-ΔF* in larvae reveals a post-embryonic role for *ago* in restricting terminal branching. (A,B) Schematic (left) and representative photomicrograph depiction of LG lateral terminal cell branches in control (A) and *hs-Gal4:UAS-agoΔF* (B) larvae. Main LG branches are indicated with black arrows, extra branches in *hs-Gal4:UAS-agoΔF* are indicated with white arrows. (C) Quantification of the number of branches per LH terminal cell in the indicated genotypes before (left) and after (right) heat shock treatment (* p<3.0×10^−14^ relative to heat shocked control).

Although the *ago^Δ3–7/1^* larval phenotypes are reminiscent of hypoxia-induced tracheal growth, they do not exclude the possibility that an earlier developmental requirement for *ago* (e.g. in the embryo) affects later branching events in the larva. To test the temporal requirement for *ago* in regulating tracheal terminal branching patterns, a dominant negative *ago* transgene (*UAS-agoΔF*) [47], [50] was combined with the *hs-Gal4* driver to produce animals in which *ago* activity could be antagonized at later developmental stages by application of a heat-shock. Whereas control and *hs>agoΔF* larvae show similar LH cell branch number prior to transgene induction (22.2±0.89 branches [n = 27] vs 21.7±0.69 branches [n = 24]), administration of a transient heat-shock to *hs>agoΔF* larvae is sufficient to drive an increase in terminal branching throughout the tracheal system (effects on LG and LH cells quantified in Figure 3A and 3B). LH cell branching is increased 24 hrs post heat-shock in *hs>agoΔF* larvae (40.2±1.48 branches [n = 24]; (p = 3.0×10^−14^ relative to no heat-shock) but remains unchanged in control larvae (22.6±0.67 branches [n = 24]). Thus animals that complete embryonic and early larval development with wt *ago* activity can be induced to undergo excess branching by transient expression of an *ago* dominant-negative allele.

**Figure 4:**
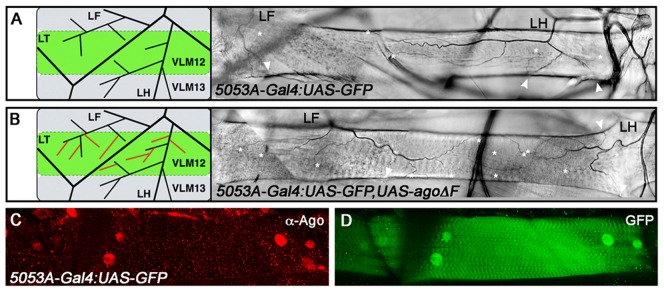
*ago* acts non-tracheal cell autonomously to regulate terminal branching. (A,B) Schematic (left) and representative photomicrograph depiction of LH and LF lateral terminal cell branch termini on VLM12 in *5053A-Gal4:UAS-nlsGFP* control (A) and *5053A-Gal4:UAS-nlsGFP,UAS-agoΔF* (B) larvae. Terminal branches terminating on VLM12 are indicated by asterisks. Arrowheads mark terminal branches with termini on other body wall muscles. (C,D) Immunofluorescence with anti-Ago antisera shows that Ago is expressed in nuclei of VLM12 (C) as marked by expression of nlsGFP (D) in *5053A-Gal4:UAS-nlsGFP* larvae.

Excess terminal branch phenotypes in *hs>agoΔF* and *ago^Δ3–7^* animals may reflect a requirement for *ago* in either tracheal or non-tracheal cell types. To directly test whether *ago* activity is required in non-tracheal tissue to restrict branching, the *agoΔF* transgene was driven with the *5053A*-*Gal4* driver (*5053A>agoΔF*), which is expressed specifically in ventrolateral body wall muscle 12 (VLM12) and has been used to study non-cell autonomous tracheogenic activity of the Btl/Bnl pathway [38]. The VLM12 muscle expresses endogenous, nuclear Ago protein (Figure 4C–4D) and is normally tracheated by the LF and LH cells (Figure 4A). The *5053A*>*agoΔF* genotype approximately doubles the number of LF and LH tracheal branches that terminate on VLM12 (Figure 4B) relative to either the adjacent muscle (VLM13) or to control larvae expressing a nuclear-localized GFP (nlsGFP) (5.11±0.16 branches [n = 54] in control vs 9.54±0.29 branches [n = 50] in *agoΔF*, p = 4.67×10^−24^) (Table 3). This degree of excess branching produced by muscle-specific expression of the *agoΔF* transgene is similar to that produced by organism-wide depletion of the Ago-RC isoform with the *ago^Δ3–7^* genomic allele. These combined genetic data provide evidence that Ago is required within larval body wall muscle cells to restrict the post-embryonic branching of nearby tracheal terminal cells.

**Table 3:** Number of tracheal terminal branches terminating on VLM12.

Genotype	# branches (± SEM)	n
*5053A-Gal4:UAS-nlsGFP*	5.11±0.16	54
*5053A-Gal4:UAS-nlsGFP,UAS-agoΔF*	9.54±0.29^a^	50
*5053A-Gal4:UAS-nlsGFP,UAS-trh*	4.97±0.25	31
*5053A-Gal4:UAS-nlsGFP,UAS-dMyc*	4.86±0.25	50
*5053A-Gal4:UAS-nlsGFP,UAS-CycE*	7.39±0.49a,b	18
*5053A-Gal4:UAS-nlsGFP,UAS-N*	4.27±0.41	15
*5053A-Gal4:UAS-nlsGFP,UAS-agoΔF,sima^07607^/+*	6.34±0.27^b^	53
*5053A-Gal4:UAS-nlsGFP,UAS-agoΔF,bnl^P1^/+*	6.54±0.28^b^	54
*5053A-Gal4:UAS-nlsGFP,UAS-Adf1^RNAi^*	5.28±0.20	40
*5053A-Gal4:UAS-nlsGFP,UAS-dVHL^RNAi^*	7.48±0.21^c^	89
*5053A-Gal4:UAS-nlsGFP,UAS-sima*	13.67±0.61^a^	48
*5053A-Gal4:UAS-nlsGFP,UAS-agoΔF,UAS-dVHL^RNAi^*	11.42±0.36b,d	52
*5053A-Gal4:UAS-nlsGFP,UAS-agoΔF,UAS-dVHL*	6.60±0.25^b^	55
*5053A-Gal4:UAS-nlsGFP,UAS-dVHL^RNAi^,UAS-ago*	6.29±0.22^d^	52

p<0.001 relative to

a *5053A-Gal4:UAS-nlsGFP*,

b *5053A-Gal4:UAS-nlsGFP,UAS-agoΔF*,

c *5053A-Gal4:UAS-nlsGFP,UAS-Adf1^RNAi^*, or

d *5053A-Gal4:UAS-nlsGFP,UAS-dVHL^RNAi^*.

### Muscle expression of Ago targets

*ago* mutations lead to tissue-specific activation of factors normally degraded by the SCF-Ago ubiquitin ligase, including the proliferative proteins CycE and dMyc in larval imaginal discs [49], [50] and the transcription factor Trachealess in tracheal cells [47]. Although the expression patterns of these proteins in body wall muscle are not well defined, we wished to test whether ectopic expression of CycE, dMyc, Trh, or the SCF-Fbw7 target Notch [reviewed in 51] was even capable of conferring a non-cell autonomous tracheogenic activity to VLM12. To this end, each of these factors was individually overexpressed using the *5053A-Gal4* driver (Table 3). Muscle-specific expression of *trh* or *Notch* failed to stimulate excess terminal branch growth. The inability of *trh* to affect tracheal recruitment to VLM12 contrasts with its ability to phenocopy *ago* mutant phenotypes in the embryonic trachea [47] and further suggests that the *ago* larval tracheal role is from separable from its embryonic role. Muscle-specific expression of *dMyc* also had no effect on the degree of terminal cell branching, despite a 28.5% increase in the 2-dimensional size of the VLM12 muscle (Table 4). Notably, increased tracheation of VLM12 driven by *agoΔF* occurs without an increase in the size of the VLM12 muscle, which is consistent with no role for post-mitotic growth in this phenotype (Table 4). *5053A>cycE* does increase terminal branch number, although to a lesser degree than *agoΔF*. However, CycE protein levels are not obviously affected by expression of *agoΔF* (Figure S1), suggesting that deregulated CycE is an unlikely cause of the non-autonomous effect of *ago* alleles on terminal cell branching.

**Table 4:** Effects of *ago* and *dMyc* on VLM12 size.^a^

Genotype	VLM12 size (pixels ± SEM)	N
*5053A-Gal4:UAS-nlsGFP*	5811.6±186.60	22
*5053A-Gal4:UAS-nlsGFP,UAS-agoΔF*	5585.3±134.00	25
*5053A-Gal4:UAS-nlsGFP,UAS-dMyc*	7470.7±262.00^b^	22

a size calculated by pixel counts using Photoshop

b p<1.0×10^−4^ relative to *5053A-Gal4:UAS-nlsGFP*.

### *ago* restricts dHIF activity and *bnl* expression in larvae

**Figure 5:**
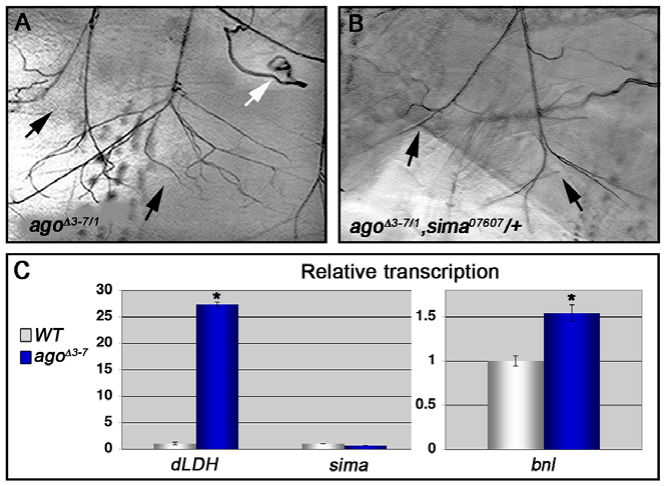
Loss of *ago* deregulates Sima activity and increases Bnl protein levels. (A,B) Representative images of LG lateral terminal cell branching in *ago^Δ3–7/1^* (A) and *ago^Δ3–7/1^*, *sima^07607^/+* (B) larvae. The main LG branches are indicated with black arrows. (C, left panel) qRT-PCR quantification of *dLDH* (left) and *sima* (right) transcription in the body wall muscles of larvae of the indicated genotypes (*p<0.001 relative to control). (C, right panel) qRT-PCR quantification of *bnl* transcription in the body wall muscles of larvae of the indicated genotypes (*p<3.2×10^−2^ relative to control).

The similarity of *ago* mutant terminal branching phenotypes to those induced by hypoxia suggests that *ago* may antagonize the dHIF pathway. To test the genetic relationship between *ago* and *sima* in larval tracheal branching, the *sima^07607^* loss-of-function allele [23] was introduced into the *5053A>agoΔF* and *ago^Δ3–7/1^* genetic backgrounds. Heterozygosity for *sima* (i.e. *sima^07607^/+*) dominantly suppressed the *agoΔF* VLM12 phenotype (Table 3) by decreasing terminal branch number from 9.54±0.29 (n = 50) to 6.34±0.27 branches (n = 53, p = 4.36×10^−12^), and also suppressed the excess and overlapping terminal branching seen in *ago^Δ3–7/1^* larvae (Table 2 and Figure 5A–5B; white arrow in 5A indicates a ringlet-shaped ganglionic branch) from 39.5±1.59 (n = 34) to 29.0±1.48 branches per LH cell (n = 34, p = 7.45×10^−6^). In addition, the *sima^07607^* allele dominantly delayed the lethal phase of both *ago^Δ3–7^* homozygotes and *ago^Δ3–7/1^* or *ago^Δ3–7/3^ trans*-heterozygotes (Table 1). Reciprocally, ectopic expression of *sima* in the VLM12 muscle (*5053A>sima*) increased tracheal recruitment in normoxic conditions (Table 3). Muscle cells are thus distinct from ectodermal cells, which do not recruit branching following overexpression of *sima* [22].

To more directly assess dHIF activity in *ago* mutant animals, the transcription of the *Drosophila LDH* gene (*dLDH*) was measured in the body wall muscle of *ago^Δ3–7^* and control larvae. *LDH* is a well-validated HIF target in vertebrates and invertebrates, and HIF-responsive elements from the *LDH* promoter have been used as the basis of HIF activity reporters in many different systems including *Drosophila* [e.g. 24]. This analysis showed a 27.3-fold increase in *dLDH* transcription in *ago* mutant larval body wall muscle preparations but no equivalent upregulation in steady-state levels of the *sima* mRNA (Figure 5C). Sima-driven expression of the FGF ligand *bnl* is a key element of the hypoxic response among non-tracheal cells [22], [38], [52]. The *bnl^P1^* loss-of-function allele dominantly suppressed both the *5053A>agoΔF* VLM12 phenotype (Table 3), from 9.54±0.29 (n = 50) to 6.54±0.28 branches (n = 54, p = 5.99×10^−11^) and the *ago^Δ3–7/1^* excess branching phenotype (Table 2), from 39.5±1.59 (n = 34) to 28.4±1.80 branches per LH cell (n = 29, p = 1.75×10^−5^). In parallel, qRT-PCR detected an ∼50% upregulation of *bnl* transcription in body wall muscle of *ago^Δ3–7^* larvae relative to control muscle (Figure 5C). Previous studies using a genomic duplication of the *bnl* locus have demonstrated that a similar 50% increase in *bnl* gene-dosage is sufficient to elicit excess tracheal terminal cell branching [38]. Thus reduced *ago* function in body wall muscle is associated with ectopic expression of the dHIF target *dLDH*, increased levels of the *bnl* mRNA, and a genetic dependence on *sima* and *bnl*.

### *ago* acts with *dVHL* to restrict tracheal terminal branching

**Figure 6:**
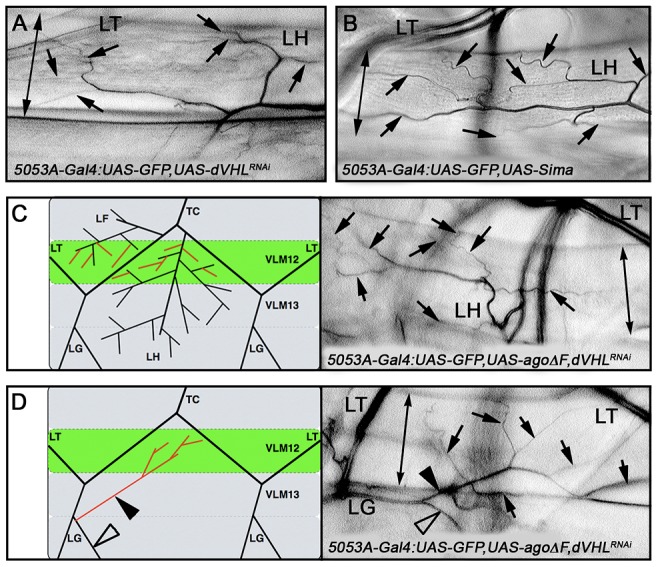
*ago* and *dVHL* co-regulate terminal branching. (A,B) VLM12-specific expression of *dVHL* dsRNA (A) or *sima* (B) leads to an increase in terminal branch number (black arrows). Double-headed arrow indicates dimension of VLM12. (C,D) Terminal branch phenotypes of *agoΔF* and *dVHL* RNAi double-mutant larvae. (C) Schematic (left) and representative photomicrograph showing LF and LH cell terminal branches (black arrows) on VLM12. Double-headed arrow indicates VLM12. (D) Schematic (left) and representative photomicrograph showing ectopic recruitment of LG cell terminal branches to VLM12 (black arrows). Black arrowhead, ectopic LG branch recruited to VLM12; open arrowhead, LG branch following its typical course; double-headed arrow indicates VLM12.

The data above suggests that *ago* alleles might exhibit functional interactions with components of the Fga/dVHL pathway, which controls Sima stability and activity *in vivo* [21]–[23], [53], [54]. A previously characterized *dVHL* RNAi knockdown transgene (*dVHL^i^*) [48]) was used with the *5053A-Gal4* driver to reduce dVHL expression in VLM12. Consistent with the role of *dVHL* upstream of *sima*, the *5053A>dVHL^i^* genotype showed an increase in terminal branching relative to a non-specific RNAi control (Figure 6A, and Table 3) (5.28±0.20 branches [n = 40] in *Adf1^i^* control vs 7.48±0.21 branches [n = 89] in *dVHL^i^*, p = 1.27×10^−9^, Figure 6). The *dVHL^i^* and *agoΔF* transgenes were then co-expressed with *5053A-Gal4* to determine their ability to enhance VLM12 tracheogenic activity (Figure 6C–6D). The *5053A>agoΔF,VHL^i^* compound genotype shows a synergistic increase in the number of branches that terminate on VLM12 (Table 3), but also leads to a phenotype not seen in either individual genotype: whereas expression of *agoΔF* or *dVHL^i^* individually increase terminal branching of LF and LH onto VLM12, the *agoΔF+dVHL^i^* combination also recruits ectopic branches from the LG lateral terminal cell (as seen in the two different focal planes of a single *agoΔF+dVHL^i^*-expressing VLM12 muscle; Figure 6C–6D) which normally bypasses VLM12. This ectopic LG recruitment phenotype occurs in approximately 10% of *agoΔF+dVHL^i^* VLM12 muscles and is also observed upon *5053A-Gal4* driven expression of *bnl* [38] or *sima* (data not shown). Thus *dVHL* and *ago* are individually required to restrict the ability of muscle cells to recruit new branch growth, and combined reduction of *ago* and *dVHL* activity leads to increased tracheogenic signals emanating from body wall muscle.

To further define the relationship between *ago* and *dVHL* in terminal branching, transgenes expressing each factor were tested for rescue of VLM12-branching phenotypes produced by reducing the function of the other (Table 3). Expression of wild type *dVHL* led to a 66% suppression of the *agoΔF* branching phenotype (p = 6.55×10^−12^); reciprocally, over-expression of wild type *ago* showed a 54% suppression of the *dVHL^i^* branching phenotype (p = 2.73×10^−4^). Thus, each gene can to some degree ameliorate non-autonomous branching phenotypes produced by loss of the other in the VLM12 segment.

### Ago controls transcriptional and organismal responses to hypoxia

**Figure 7:**
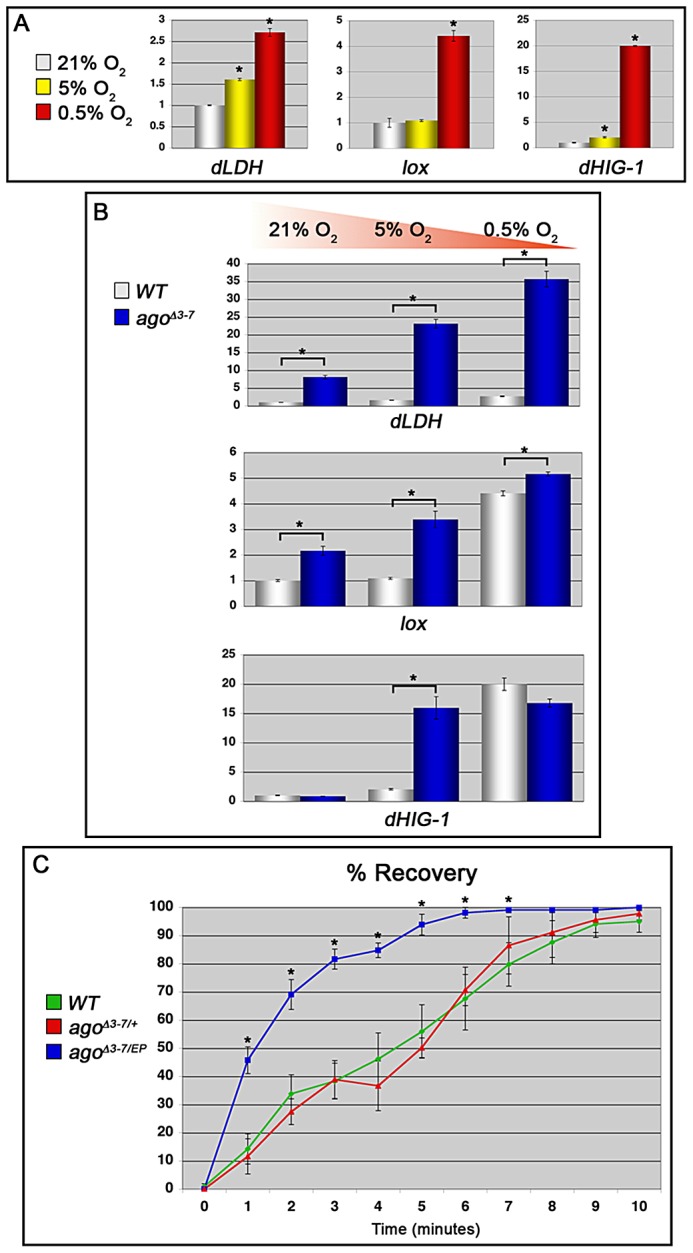
*ago* loss alters the transcriptional and organismal response to hypoxia. (A) Quantification of *dLDH* (left), *lox* (center) and *dHIG1* (right) transcription in control larvae in the indicated conditions by qRT-PCR (*p<0.01). (B) Quantification of *dLDH* (top), *lox* (middle) and *dHIG1* (bottom) transcription in the indicated genotypes by qRT-PCR in 21% (left), 5% (center) and 0.5% (right) oxygen (*p<0.01). (C) Quantification of post-hypoxic recovery time in adult *Drosophila* of the indicated gentoypes following acute hypoxia (*p<0.05 relative to control).

In view of the genetic and molecular links between *ago*, *sima*, *dLDH*, and *dVHL*, the organism-wide transcriptional response to hypoxia was examined in *ago* mutant animals. *Drosophila* respond to varying degrees of hypoxia by driving transcription of distinct sets of target genes at differing oxygen concentrations, including those involved in metabolic adaptation and survival in low oxygen [31], [32]. A subset of hypoxia-inducible genes was selected for this analysis based on their differential transcription in hypoxic adult *Drosophila* [31] and predicted links to known mechanisms of the hypoxic response. These included *dLDH*, which plays a role in the metabolic switch to high flux glycolysis [reviewed in 55], [56], *lysyl oxidase* (*lox*), a HIF target in mammalian cells that plays a role in hypoxia-induced changes in cell adhesion [57], [58] and vascular remodeling [59], and *dHIG1* (*CG11825*), the *Drosophila* homolog of Hypoxia Induced Gene-1 (HIG1), which is induced by HIF and promotes cell survival [60]. qRT-PCR analysis was carried out for each of these genes under conditions of decreasing environmental oxygen (21%, 5%, 0.5%) in whole control larvae or whole *ago^Δ3–7^* larvae (Figure 7A–7B). We find that each of these genes is differentially induced in hypoxia in a manner consistent with findings in adult *Drosophila* [31] and can be ectopically induced by the *ago^Δ3–7^* allele. *dLDH* is minimally transcribed in normoxic control larvae, and with progressively higher transcription as the oxygen level falls (1.6 and 2.7-fold increases in 5% and 0.5% O_2_ respectively, Figure 7A), confirming that *dLDH* transcription increases with increasing dHIF activity. In *ago^Δ3–7^* homozygous animals, *dLDH* expression is increased 8.1-fold in whole normoxic larvae (this lower fold induction in the whole larva relative to the ∼27-fold enrichment seen in dissected body wall muscle in Figure 5C is presumably a reflection of the tissue-specific nature of the *ago^Δ3–7^* allele), and is increased approximately 14-fold in *ago^Δ3–7/1^* larvae relative to control larvae at both 5% and 0.5% O_2_ (Figure 7B, top panel). Thus *ago* restricts *dLDH* expression activity across a broad range of oxygen concentrations. The *lox* gene is normally only up-regulated in whole control larvae by strong hypoxia (4.4-fold induction at 0.5% O_2_; Figure 7B). The *ago^Δ3–7^* allele leads to a 2.2-fold increase in *lox* transcription in normoxia, and *lox* transcription reaches near maximal levels at 5% O_2_; the 3.4-fold induction seen in *ago* mutants in 5% O_2_ is not significantly different from that seen in control larvae at 0.5% O_2_ (Figure 7B, middle panel). This pattern suggests that the *lox* promoter is induced by levels of dHIF activity achieved in moderate hypoxic conditions, and that this threshold is more easily reached in *ago* mutants. The *dHIG1* gene displays a more exaggerated version of the *lox* response pattern: *dHIG1* mRNA levels are only induced strongly (19.9-fold) in whole control larvae by 0.5% O_2_ (Figure 7A); the *ago^Δ3–7^* allele is not sufficient to drive ectopic *dHIG1* transcription in normoxic conditions but it is sufficient to sensitize the *dHIG1* promoter to reduced O_2_ levels such that maximal *dHIG1* expression is now achieved at a ten-fold higher O_2_ concentration than normal (Figure 7B, bottom panel). In addition to *dLDH*, *lox*, and *dHIG1*, three other genes also induced by hypoxia, *hairy*, *amy-p* and *thor* genes [31], are also moderately up-regulated in normoxic *ago^Δ3–7^* mutant larvae (Table S1). Reducing *ago* activity is thus sufficient to alter the threshold required to drive expression of multiple hypoxia-inducible genes.

Adult *Drosophila* respond to prolonged periods of oxygen deprivation by entering into a state of hypoxic stupor characterized by inactivity and reduced oxygen consumption [34]. Many mutations have been identified that slow this hypoxic recovery [32], [33], [35], but few mutations have been described that enhance it. Under our standard laboratory conditions, control adult flies enter stupor after approximately fifteen to twenty minutes in a 0.5% O_2_ environment and remain unconscious until re-oxygenation. We assayed control *+/+* adults, *ago^Δ3–7^/+* adults, and adults *trans*-heterozygous for the *ago^Δ3–7^* allele and the *ago* hypomorphic allele *EP(3)1135* (BDSC) for recovery time following acute hypoxia (1 hour at 0.5% O_2_) (Figure 7C). *ago^Δ3–7^/EP(3)1135* flies display no obvious developmental phenotypes and enter into hypoxic stupor at the same rate as control flies (data not shown); however, they recover significantly faster than either control *+/+* or *ago^Δ3–7^/+* adults. Linear regression analysis indicates that the time for 50% recovery is reduced from 4.5±0.75 minutes in control *+/+* flies, to 1.4±0.16 minutes in *ago^Δ3–7^/EP(3)1135* flies (p = 0.0015). The *ago^Δ3–7^/EP(3)1135* population also reaches 100% recovery after 10 minutes of re-oxygenation, whereas neither the control *+/+* or *ago^Δ3–7^/+* populations achieved 100% recovery by the end of the 15 minute measurement period (data not shown). Thus, the genetic evidence of a role for *ago* as a regulator of dHIF-regulated branching in the larval tracheal arbor is paralleled at the organismal level by an enhanced transcriptional sensitivity to hypoxia and an increased ability of flies to recover from a transient hypoxic challenge.

### Ago associates with Sima and suppresses Sima levels

**Figure 8:**
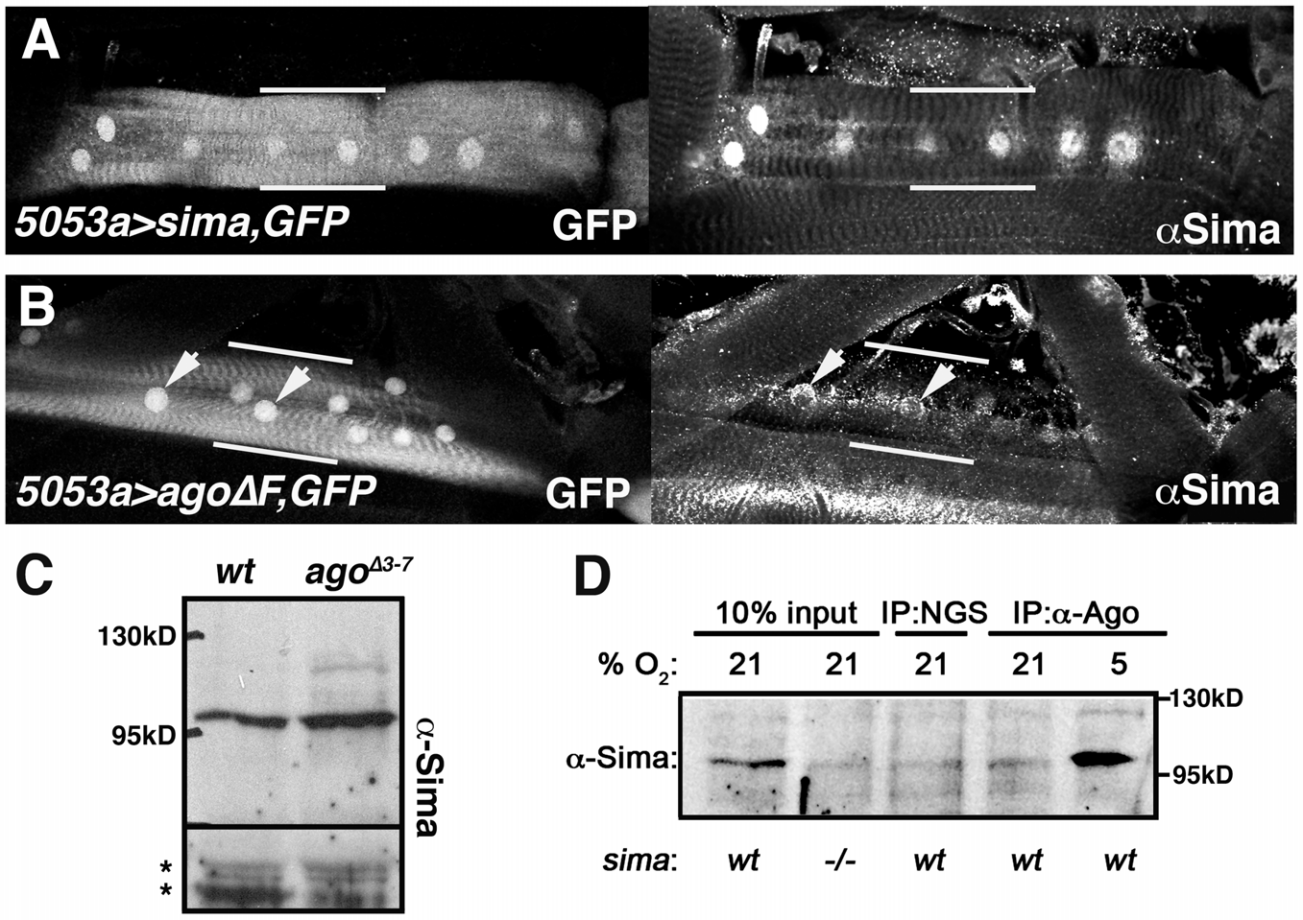
Ago associates with Sima and limits its abundance in peripheral tissues. Confocal images of GFP and anti-Sima fluorescence in (A) *5053A>sima,nlsGFP* and (B) *5053A>agoΔF,nlsGFP* larval muscles. Nuclear-enriched GFP marks the VLM12 muscle (bracketed by white bars) in the field of surrounding body wall muscle segments. Arrows in (B) indicate Sima-positive nuclei. (C) Anti-Sima immunoblot of wt and *ago^Δ3–7^* 3^rd^ instar filleted pelts. Molecular weight (MW) markers are indicated. Asterisks denote non-specific bands to indicate loading. (D) Immunoblot of Sima in control (*wt*) and *sima^07607^* (*−/−*) whole larval lysates (lanes 1–2), and in normal goat serum (NGS) and anti-Ago immunoprecipitates from normoxic (21% O_2_) or hypoxic (5% O_2_) larvae.

To test the molecular relationship between Sima and Ago, Sima levels were assessed in two ways: by immunoflourescent staining of VLM12 muscles expressing the *UAS-agoΔF* transgene and by Western blotting of lysates of *ago^Δ3–7^* larvae (Figure 8). Fluorescence microscopy confirms that a previously described anti-Sima antibody [24] detects high levels of transgenically expressed Sima in the VLM12 nuclei of *5053A>sima* muscles, and that endogenous Sima is not readily detectable by this method of analysis in the nuclei of adjacent non-transgenic muscles (Figure 8A). Following expression of the *agoΔF* dominant-negative transgene (*5053A>agoΔF*), a fraction of VLM12 nuclei accumulate anti-Sima reactive epitopes (see arrows, Figure 8B). This same anti-Sima antibody detects elevated levels of an ∼110 kD molecular weight band in *ago^Δ3–7^* filleted 3^rd^ instar pelts relative to *wt* control pelts (Figure 8C). This ∼110 kD band is absent in lysates of *sima^07607^* larvae (Figure 8D, lane 1 vs. 2), and is specifically enriched in precipitates of an anti-Ago polyclonal antibody from lysates of hypoxic larvae (Figure 8D, lane 5). Collectively, these molecular data indicate that Ago can associate with Sima in larval lysates, and that Ago limits Sima levels in developing tissues.

## Discussion

The selective stabilization of the Sima/HIF–1α transcription factor in hypoxia plays a key role in the response of metazoan organisms to low oxygen concentrations by its ability to induce a program of hypoxia-specific gene expression [reviewed in 2]. Evidence suggests that in *Drosophila*, Sima plays a dual role in the post-mitotic growth of tracheal terminal branch cells toward hypoxic peripheral tissues by acting within both the ‘signaling’ hypoxic peripheral cells and in the ‘responsive’ terminal tips cells [reviewed in 37].

Our data implicate the Ago-SCF ubiquitin ligase as a required regulator of Sima during hypoxia sensing in peripheral cells, but do not rule out an additional role for Ago within tracheal terminal tip cells which contributes to their ectopic branching in *ago* mutant larvae (see below). Phenotypes produced by muscle-specific expression of an *ago* dominant-negative allele, or by a genomic allele that specifically affects *ago* expression in peripheral tissues, are phenocopied by overexpression of Sima (this study) or the FGF homolog Bnl [38]. These non-cell autonomous effects of *ago* alleles on terminal branching are accompanied by a strong induction in peripheral tissues of the dHIF target *dLDH*, and can be dominantly suppressed by an allele of *sima*. *ago* alleles induce expression of a set of dHIF-inducible hypoxia-response genes in normoxia that includes *dLDH*, and this is paralleled at the organismal level by an enhanced ability of *ago* mutant flies to the recover from hypoxic stupor. *ago* alleles are thus among the first genetic alterations shown to enhance the recovery of adult *Drosophila* from hypoxic exposure. Within larval muscle, *ago* appears to inhibit *sima* in parallel to *dVHL*, which targets the Sima/HIF–1α protein for constitutive degradation in normoxia [reviewed in 3]. Consistent with this, we find evidence that Ago can associate with Sima and limits its levels *in vivo*. Collectively these data significantly expand the known role of Ago in organism development by demonstrating that it is required in an apparently novel pathway that collaborates with dVHL to inhibit Sima-regulated hypoxic gene expression in peripheral tissues.

Though the work presented here focuses on the ‘tracheo-attractant’ effects of reducing *ago* expression in body wall muscle, this may be just one manifestation of roles Ago plays in controlling hypoxia-regulated gene expression. Indeed, reducing *ago* function has a quantitatively stronger effect on terminal branching than a genomic duplication of the *bnl* locus [38], suggesting either that *ago* also act within tracheal cells to limit branching [as in 47], [48] or that a larger set of dHIF target genes contribute to the effect. Consistent with this latter hypothesis, normoxic *ago* mutant larvae display ectopic induction of hypoxia-responsive metabolic genes such as *dLDH*, *lox*, *hairy*, *amy-p* and *thor*. Based on this profile, it appears that *ago* mutant larvae reared in normoxia elevate expression of *bnl* but also engage a metabolic switch to high-flux glycolysis that is characteristic of hypoxic cells [32], [33], [35], [36], [61]. Future studies will be required to assess the full effect of these transcriptional changes on the behavior of terminal tracheal cells and the tissues into which they project.

In wild type animals, the transcriptional response of cells to hypoxia is graded such that different target genes are induced across a range of environmental O_2_ concentrations [31]. In *ago* mutants, this differential induction is largely abolished such that expression of genes such as *lox* and *dHIG1* is virtually indistinguishable at 5% and 0.5% O_2_. Thus, *ago* appears to be required both for inhibition of hypoxia-inducible genes in normoxia and for the graded expression of hypoxia-inducible genes under variable levels of oxygen deprivation. We hypothesize that this graded sensitivity is normally a product of the interaction between the Ago and Fga/dVHL regulatory mechanisms. The HPH/VHL pathway has been demonstrated to act in a graded manner, such that it degrades HIF–1α efficiently in normoxia, but is progressively less efficient as the oxygen concentration drops [62]. This leads to a gradient of HIF activity that is presumably required for the differential induction of target genes. We hypothesize that *ago* acts in parallel to *dVHL* to dampen Sima/HIF-1 activity across a range O_2_ concentrations, and that Ago may function as a dHIF regulatory mechanism at very low O_2_ concentrations in which the HPH/dVHL pathway is hypothesized to be inactive [62]. Thus, the absence of Ago allows a mild hypoxic stimulus (∼e.g. 5% O_2_) to be translated into levels of dHIF-dependent gene expression that would normally only result from much stronger hypoxic exposure. The data presented here support this prediction, with the end result that the transcriptional response profile of hypoxia-response genes in *ago* mutant larvae is shifted toward induction by more mild stimuli.

The molecular mechanism(s) underlying the genetic relationship between *ago* and *sima* in tracheal branching appears to involve a physical association of their encoded proteins that modulates Sima levels. Given that Ago is a ubiquitin ligase specificity factor, these data are consistent with a model in which Ago supports Sima polyubiquitination and turnover. Recent studies have identified the human Ago ortholog Fbw7 as a HIF–1α interacting factor and have proposed that Fbw7 promotes HIF-1α turnover following GSK3ß phosphorylation in cultured cells [45], [46]. Phenotypic predictions made by this molecular model appear to be confirmed by the *ago* terminal cell branching phenotypes documented here. Intriguingly, RNAi depletion of *GSK3ß/shaggy* or a proteasome subunit in VLM12 also elevates the number of tracheal branches that terminate on this muscle (Table S2). However Fbw7 is implicated in the proteolytic destruction of two transcription factors, the Notch intracellular domain (NICD) [reviewed in 51] and sterol-regulatory enhancer binding protein (SREBP) [63], that indirectly modulate HIF-dependent hypoxic gene expression in eukaryotic cells [64], [65]. Ago could thus theoretically influence hypoxic gene expression via these paths as well. Future biochemical studies will be required to clarify the full range of Ago molecular targets that contribute to its role in hypoxic gene expression.

The well-studied anti-proliferative role of *ago* is conserved in its human ortholog *Fbw7*, which is mutationally inactivated in a wide spectrum of primary human cancers [reviewed in 51]. Some cancer cells engage a program of gene expression that supports a switch to high-flux glycolysis (a phenomenon termed ‘Warburg effect’ [26]) and are more resistant to transient hypoxia than normal cells [reviewed in 1]. Both of these properties can now, to some degree, also be associated with *ago* loss in *Drosophila*. In view of the functional conservation between SCF-Ago and SCF-Fbw7 in degradation of shared oncogenic targets [49], [50] and the proposed role of Ago/Fbw7 in Sima HIF-1α turnover [this study and 45], [46], our data raise the interesting possibility that sensitization to mild hypoxia may be a feature of *Fbw7* mutations in vertebrates as well. If so, then tumor suppressive properties of *Fbw7* may derive in part from its established anti-proliferative role and in part due to modulation of HIF-regulated angiogenic and metabolic pathways.

## Materials and Methods

### Stocks, genetics, and statistics

The *FRT80B* and *w^1118^* strains were used as wild type controls. The *ago^1^* and *ago^3^* alleles have been previously described [49]. The *ago^Δ3–7^* allele was identified as an imprecise excision of the *ago^EP(3)1135^* transposon. Alleles used in this study: *bnl^P1^*, *sima^07607^*, *ago^EP(3)1135^* (all from the Bloomington *Drosophila* Stock Center) and *1-eve-1* [66]. The following transgenes were also used: *UAS-agoΔF* and *UAS-ago* [47]; *UAS-CycE*, *UAS-dMyc*, *UAS-N*, *UAS-nlsGFP* (all from the Bloomington *Drosophila* Stock Center), *UAS-trh* [67], *UAS-sima* [24], *UAS-dVHL* [21], *UAS-Adf1^RNAi^* and *UAS-sgg^RNAi^* (Vienna *Drosophila* RNAi Center), *UAS-dVHL^RNAi^* [48], *hs-Gal4*, and *5053A-Gal4* (from the Bloomington Drosophila Stock Center). Statistical comparisons were made using Student's t-Test (Microsoft Excel) with the indicated significance levels.

### Hypoxia treatments

Hypoxia treatments were performed in a sealed Modular incubator chamber (Billups-Rothenberg Inc., Del Mar, CA) with separate gas intake and exhaust openings. Internal O_2_ concentration was measured with an electronic O_2_ sensor (OX-01, RKI Instruments, Inc., Union City, CA). To assay recovery from hypoxia, 5–7 day old adult flies were put into plain glass tubes in groups of 9–15. The flies were then placed into the hypoxia chamber at 0.5% O_2_ for one hour and then removed to normoxia. Following hypoxic treatment, >99% of the flies (178 of 179) had fallen into hypoxic stupor. Recovery time was defined as the time required for each individual fly to resume walking following re-oxygenation.

### Reverse transcription and quantitative real-time PCR (qRT–PCR)

Total RNA was isolated from dissected third instar larval body wall muscles. cDNA was reverse-transcribed using random hexamer primers (Invitrogen) with Superscript II Reverse Transcriptase (Invitrogen). *dVHL* and *ß-tubulin* transcripts were then amplified with gene-specific primers. For quantification of mRNA levels, total RNA was isolated from whole third instar larvae or dissected larval tissues and reverse transcribed as described above. Levels of *Arp87c*, *ago*-*RA*, *-RB* and *-RC*, *dLDH*, *sima*, *bnl*, *lox*, *hairy*, *dHIG1*, *thor* and *amy*-*p* were then assayed with gene-specific primers using the SYBR green method of quantitative real-time PCR on a Roche LightCycler 480 machine. Transcript abundance was normalized to levels of *Arp87c* as in [31].

### Imaging of third instar larval trachea

To image the larval tracheal system, third instar larvae were dissected in cold PBS and fixed in 4% paraformaldehyde. Air-filled tracheal branches were imaged using bright-field microscopy. and assembled using Photomerge (Adobe Photoshop CS).

### Immunohistochemistry, Western blotting, and immunoprecipitation

Third instar larvae were dissected in cold PBS, fixed in 4% paraformaldehyde and incubated with guinea pig anti-CycE (1∶500) or rabbit anti-Sima (1∶1000). Secondary antibodies (anti-guinea pig conjugated to Cy3 or anti-rabbit conjugated Cy5) were used as recommended (Jackson ImmunoResearch). To assess Sima protein levels in third instar larvae, larval pelt extracts were prepared in sample buffer and resolved on 7.5% SDS-PAGE prior to Western blotting with rabbit anti-Sima (1∶1000) [24] and developed with anti-rabbit HRP (1∶1000; Jackson ImmunoResearch). Whole larval extracts were immunoprecipitated with guinea pig anti-Ago polyclonal sera (1∶1000) [47] prior to immunoblotting with anti-Sima antibody.

## Supporting Information

Figure S1 Loss of *ago* does not deregulate Cyclin E levels in body wall muscle cells. Comparison of Cyclin E levels in VLM12 and VLM13 in *5053A-Gal4:UAS-GFP,UAS-agoΔF* larvae. Larvae were stained with a-Cyclin E antiserum (red). GFP marks VLM12 (green).
(TIF)

Table S1 Relative induction of hypoxia-responsive genes in normoxic *ago* larvae. Relative levels of mRNAs of the indicated genes normalized to the level of each mRNA in *wt* control larvae. Experiments were done in triplicate. P-values provided for each gene.
(DOCX)

Table S2 Number of tracheal terminal branches terminating on VLM12. Number of tracheal terminal branches terminating on the VLM12 muscle segment in the indicated genotypes. P-values are indicated.
(DOCX)
