# Supplementary figures and images for: The Archipelago Ubiquitin Ligase Subunit Acts in Target Tissue to Restrict Tracheal Terminal Cell Branching and Hypoxic-Induced Gene Expression
Source: PLoS Genet. 2013 Feb 14;9(2):e1003314. doi: 10.1371/journal.pgen.1003314 (PMC3573119; doi:10.1371/journal.pgen.1003314)

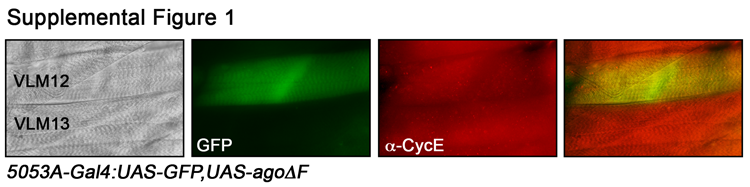

Supplement: Figure S1 — Loss of ago does not deregulate Cyclin E levels in body wall muscle cells. Comparison of Cyclin E levels in VLM12 and VLM13 in 5053A-Gal4:UAS-GFP,UAS-agoΔF larvae. Larvae were stained with a-Cyclin E antiserum (red). GFP marks VLM12 (green). (TIF) [file pgen.1003314.s001.tif]
