# Supplementary material for: The Archipelago Ubiquitin Ligase Subunit Acts in Target Tissue to Restrict Tracheal Terminal Cell Branching and Hypoxic-Induced Gene Expression
Source: PLoS Genet. 2013 Feb 14;9(2):e1003314. doi: 10.1371/journal.pgen.1003314 (PMC3573119; doi:10.1371/journal.pgen.1003314)
Supplement: Table S1 — Relative induction of hypoxia-responsive genes in normoxic ago larvae. Relative levels of mRNAs of the indicated genes normalized to the level of each mRNA in wt control larvae. Experiments were done in triplicate. P-values provided for each gene. (DOCX) [file pgen.1003314.s002.docx]

**Supplemental Table 1.**

**Relative induction of hypoxia inducible genes in normoxic ago larvae**

| **Target gene** | **Relative Transcription ^a^** | **P-value ^b^** |
| --- | --- | --- |
| *dLDH* | 27.3 ± 0.47 ^c^ | 6.06x10^-5^ |
| *dLDH* | 8.11 ± 0.52 ^d^ | 4.97x10^-4^ |
| *lox* | 2.16 ± 0.17 ^d^ | 6.38x10^-3^ |
| *hairy* | 1.19 ± 0.02 ^d^ | 1.11X10^-3^ |
| *amy-p* | 2.03 ± 0.07 ^d^ | 9.87x10^-5^ |
| *thor* | 1.18 ± 0.05 ^c^ | 2.99x10^-2^ |

^a^ Transcription normalized to *wt* control larvae; experiments done in triplicate.

^b^ P-value relative to control (Student’s t-test)

^c^ RNA prepared from body wall muscle

^d^ RNA prepared from whole larvae
