# Supplementary material for: The Archipelago Ubiquitin Ligase Subunit Acts in Target Tissue to Restrict Tracheal Terminal Cell Branching and Hypoxic-Induced Gene Expression
Source: PLoS Genet. 2013 Feb 14;9(2):e1003314. doi: 10.1371/journal.pgen.1003314 (PMC3573119; doi:10.1371/journal.pgen.1003314)
Supplement: Table S2 — Number of tracheal terminal branches terminating on VLM12. Number of tracheal terminal branches terminating on the VLM12 muscle segment in the indicated genotypes. P-values are indicated. (DOCX) [file pgen.1003314.s003.docx]

**Supplemental Table 2. Number of tracheal terminal branches terminating on VLM12.**

| **Genotype** | **# branches (± SEM)** | **n** |
| --- | --- | --- |
| *5053A-Gal4:UAS-GFP* (21°C) | 5.06 ± 0.25 | 34 |
| *5053A-Gal4:UAS-GFP,UAS-Pros26^1^,UAS-Prosß2^1^* (21°C) | 7.38 ± 0.25^a^ | 53 |
| *5053A-Gal4:UAS-GFP,UAS-Adf1^RNAi^* | 5.28 ± 0.20 | 40 |
| *5053A-Gal4:UAS-GFP,UAS-sgg^RNAi^* | 7.71 ± 0.26^b^ | 48 |

^a,b^ p < 1.0X10^-4^ relative to ^a^ *5053A-Gal4:UAS-GFP* @ 21°C, or ^b^ *5053A-Gal4:UAS-GFP,UAS-Adf1^RNAi^*.
